# Novel therapies delay the progression of smoldering multiple myeloma: Case report and discussion

**DOI:** 10.3332/ecancer.2010.182

**Published:** 2010-05-11

**Authors:** A. Jhaveri, F. Muggia

**Affiliations:** 1 Department of Medicine; 2 Division of Medical Oncology, NYU School of Medicine, New York, NY-10016, USA

## Abstract

This clinical vignette illustrates how our therapeutic approaches to early stages of multiple myeloma have changed over the past decade with novel therapies reducing disease and preventing disease progression. Recent paradigms of multiple myeloma describe the disease as a spectrum of clinical stages, including asymptomatic ‘smoldering’ states that progress to symptomatic states. The average 5-year survival rate of patients with multiple myeloma diagnosed between 1996 and 2004 according to surveillance epidemiology and end results (SEER) data is 35.9%. Here, we describe the use of novel therapeutic agents including bortezomib, lenalidomide, bisphosphonates, Doxil/Caelyx, and dexamethasone, and their success in affecting the course of disease. Multiple trials have shown an increased benefit of these newer agents over prior multiple myeloma treatment regimens. At 13 years and 8 months from diagnosis, our patient is doing well, and thus is a model of how long-term control of multiple myeloma prolongs survival.

## Case

**Figure 1:**
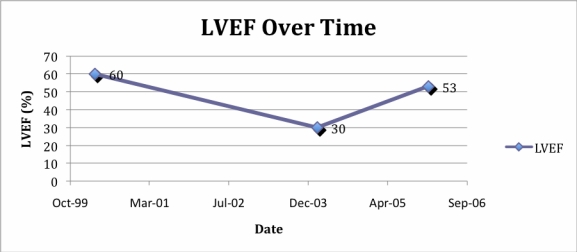
LVEF over time. A normal LVEF by echocardiography was documented in April 2000 after two cycles of free doxorubicin and at the onset of Doxil/Caelyx. The patient had no signs and symptoms of cardiac disease until bortezomib was added to Doxil/Caelyx (February 2004) and simultaneously with a marked drop in IgA. The Doxil/Caelyx was discontinued and after medical management of hypertension and heart failure, the LVEF rose towards baseline in January 2006.

A 59-year-old woman was found to have increased total protein on routine blood tests in February 1996. Serum protein electrophoresis revealed an IgA spike of 2.2 g/dl, and bone marrow biopsy showed 20%–30% plasma cells, confirming the diagnosis of multiple myeloma (because of lack of symptoms, Kyle and colleagues would classify it as ‘smoldering’ – see in the following). According to the earliest blood work available, the patient’s haemoglobin was 11.5 in December 1998. At the outset and in subsequent evaluations she never went on to demonstrate lytic bone lesions (but did eventually manifest a compression fracture of T11); likewise there was no proteinuria, and chemistries including calcium and creatinine were normal. Medications that the patient was taking at the time were enalapril, hydralazine, carvedilol, and levothyroxine, respectively. The patient was started on melphalan and dexamethasone, in large part because of concerns with the amount of paraprotein. Despite this therapy, however, her IgA rose further from greater than 2–4.1 g/dl in 1999; she subsequently underwent treatment with four cycles of carmustine, doxorubicin, and dexamethasone. In response to this regimen, her IgA fell to 1.9 g/dl. However, the course was complicated by febrile neutropenia requiring two hospitalizations and intravenous antibiotics. In early 2000, she was started on intravenous bisphosphonates (pamidronate) by her current oncologist (FM), and as her IgA again rose to 2.9 g/dl, she was started on pegylated liposomal doxorubicin (Doxil/Caelyx) 40 mg/m^2^ every 4 weeks. Her IgA declined and remained stable at 1.9 g/dl for nearly 3 years (Figure 1).

**Figure 2:**
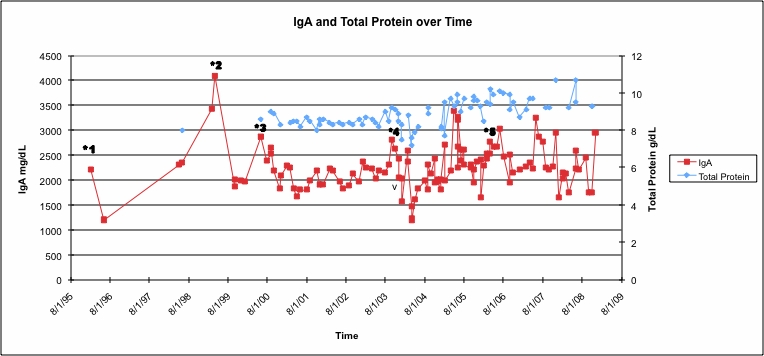
IgA and total protein over time in relation to treatments underwent by patient. The patient’s IgA has been stable between 2000 and 3000 mg/dl since the addition of bortezomib in 2003. The total protein has been stable between 8.0 and 9.0 g/dl with treatment. Treatments are starred as following: *1 = melphalan and dexamethasone; *2 = carmustine and doxorubicin; *3 = pegylated liposomal doxorubicin; *4 = bortezomib; *5 = lenalidomide added to continued bortezomib.

When the IgA again rose to 2.8 g/dl at the end of 2003 in spite of 4 months of thalidomide added to the regimen, she was begun on bortezomib that had just become available. The addition of bortezomib 1.3 mg/m^3^ days 1, 4, 8, 11 every three weeks to Doxil/Caelyx and pamidronate brought the IgA promptly down to 1.5 g/dl. However, the patient experienced symptoms of congestive heart failure (venous distension, fatigue, and cardiac enlargement by X-ray). The left ventricular ejection fraction on transthoracic echocardiography was 30%, and Doxil/Caelyx was discontinued (Figure 1). Bortezomib was reduced to 2.2 mg on days 1, 4, 8 every 21 days in accordance to patient wishes. On bortezomib, the patient’s IgA remained stable at 1.8 g/dl for about two years until rising slowly accompanied by an increase in serum creatinine to above the normal range. These events prompted the addition of the newly approved lenalidomide (in doses no higher than 10 mg daily x 10 days, because of her prior intolerance to thalidomide) to bortezomib in May 2006. The patient’s IgA has subsequently been stable at 2.2 g/dl in June 2008 on this combination, and her creatinine has returned towards normal (Figure 2). She has also been continued on zoledronate every 3 months. She is clinically well except for having some progressive inability to walk up stairs and dyspnoea on exertion in spite of a maximal regimen for cardiac pre-load and after-load reduction. Bortezomib has been attenuated to treatment on days 1 and 4 every 4 weeks, in part, because of the development of mild but recurrent gastrointestinal symptoms and recent complaints of grade 1 sensory neuropathy.

## Discussion

Multiple myeloma is a malignant proliferation of plasma cells and accounts for 1% of all malignancies. This disease affects one to five individuals per 100,000 [1]. The average 5-year survival rate of patients with multiple myeloma that was diagnosed between 1996 and 2004 according to surveillance epidemiology and end results (SEER) data is 35.9% [2]. Survival for patients with symptomatic multiple myeloma is 3–4 years.

The above clinical vignette illustrates advances made in multiple myeloma treatment and understanding over the past 15 years and their effects on improving outcomes and survival in earlier stages of myeloma as shown in the aforementioned patient. In current paradigms of multiple myeloma, multiple myeloma arises from an asymptomatic premalignant disorder that is known as monoclonal gammopathy of unknown significant (MGUS). MGUS first progresses to ‘smoldering’ multiple myeloma, an asymptomatic state, and then to symptomatic multiple myeloma. The International Myeloma Working Group defines smoldering myeloma as a disorder in which the patient has a serum monoclonal protein level of 3 g/dl or more or a proportion of plasma cells in the bone marrow of 10% or more with no end-organ damage (hypercalcemia, renal insufficiency, anaemia, bone lesions, recurrent bacterial infections, etc.) [3]. Patients with smoldering myeloma have a 73% chance in 15 years in progressing to multiple myeloma, and this risk is greatest in the first few years after diagnosis [4]. The median time to progression to symptomatic disease is 1–2 years [5].

Southwest Oncology Group sequential studies of treatments for symptomatic multiple myeloma from 1970s well into the 1990s showed little progress with the addition of multiple chemotherapeutic drugs over what could be achieved with alkylating drugs such as melphalan in combination with glucocorticoids [6,7]. However, the introduction of bisphosphonates and thalidomide heralded changes in outcome wrought outside the traditional cytotoxic drugs [8,9]. These changes were followed by the dramatic activity of a proteasome inhibitor, bortezomib, and later by a better tolerated immunomodulatory drug, lenalidomide, to replace thalidomide [10,11]. Understanding of the molecular biology of the malignant plasma cell and its relationship to the bone marrow stroma is leading to several additional advances in treatment strategies.

Although prior recommendations suggested close follow-up for patients with smoldering myeloma, new trials have been studying benefits in using new therapies for this group of patients in an effort to improve survival or even prevent progression to symptomatic disease. The recommendation for observation of asymptomatic patients is based on trials of asymptomatic patients randomized to observation versus melphalan and prednisone therapy. These studies showed no differences in survival. However, new agents may change this paradigm [12,13]. Investigators have been studying the use of bisphosphonates, thalidomide, lenalidomide, and bortezomib in the treatment of smoldering myeloma. In the context of the current patient, bortezomib, lenalidomide, bisphosphonates, Doxil/Caelyx, and dexamethasone appeared to affect the course of the disease more than the initial chemotherapy.

Thalidomide, an anti-angiogenic agent, may delay progression to active disease. Early trials with thalidomide were based on the observation of increased angiogenesis in multiple myeloma [14]. Rajkumar et al. showed that thalidomide is effective in treating early stage multiple myeloma [15]. One trial of thalidomide in early stage multiple myeloma found a 63% 2-year progression-free survival. In this study, 34% of study participants had a partial response to thalidomide therapy [16]. Another study found a median survival of more than 5 years with thalidomide therapy for smoldering multiple myeloma [17].

Lenalidomide is a third generation immunomodulatory drug that is a thalidomide analogue but with improved side effects. Although studies have shown high clinical responses and survival benefits of lenalidomide in newly diagnosed, untreated myeloma, further studies need to be performed to assess its effectiveness in asymptomatic myeloma [18,19]. In patients with newly diagnosed multiple myeloma, initial treatment with a regimen of lenalidomide, melphalan, and prednisone had a 100% survival at 1 year, and an initial treatment combination of dexamethasone and lenalidomide showed a 3-year survival of 88% [20,21]. These overall survival results are significantly better than that for the prior gold standard regimen of melphalan and prednisone.

Bortezomib is the first proteasome inhibitor to be used in clinical treatment and represents a novel approach to cancer therapy. By inhibiting the ubiquitin-proteasome signalling pathway, it leads to cell death. Although bortezomib was first studied and found to be effective in patients with refractory or relapsed myeloma, it has also been shown to be effective in previously untreated patients [22]. The Phase III Vista trial showed that in elderly patients with previously untreated myeloma not eligible for transplantation, the addition of bortezomib to the previous gold standard of melphalan and prednisone improved both length of time before disease progression and overall survival. The complete response rate of bortezomib, melphalan, and prednisone was 30%, while it was 4% in the control group receiving melphalan and prednisone. The median time to progression was 24 months in the group with bortezomib, while it was 16.6 months in the control group [23–25]. More recently, the combination of bortezomib with Doxil/Caelyx was found to be effective and gained FDA approval [26,27]. This combination has been very well tolerated, although in our patient, it was associated with perhaps coincidental acute cardiac decompensation. The aetiology of our patient’s cardiac disease is not clear, and is associated with hypertension, unlike anthracycline cardiomyopathy [28].

Bisphosphonates are now an integral part of the therapeutic armamentarium, not only in preventing the progression of bone disease and pathologic fractures, but perhaps also working via the microenvironment to decrease the proliferation and invasiveness of the myeloma cells. Better supportive care and prevention of complications associated with multiple myeloma such as hypercalcemia, renal failure, and bone lesions have also contributed to improved survival and quality of life. New therapies as described above may delay or even prevent the progression of smoldering multiple myeloma to symptomatic states. Advances in both clinical care and therapeutic agents are allowing patients to live longer with this disease without compromising quality of life in a major way. The course in this particular patient is illustrative of how long term control of the disease is achievable by the introduction of a number of measures associated with an excellent therapeutic index.
